# Sanitation of medical cannabis flowers (*Cannabis sativa L.*, flos): comparing current technologies and non-thermal plasma novelties from safety and quality perspectives

**DOI:** 10.1186/s42238-026-00417-9

**Published:** 2026-03-12

**Authors:** Samantha Nestel, Claas Hedtfeld, Ulrike Spilker, Jörg Ehlbeck, Sebastian Guenther, Uta Schnabel

**Affiliations:** 1 Leibniz Institute for Plasma Science and Technology e.V. (INP), Felix-Hausdorff-Str. 2, Greifswald, 17489 Germany; 2 HerbaMedica GmbH, Fanny-Zobel Str. 9, Berlin, 12435 Germany; 3 Institute of Pharmacy, Universität Greifswald, Friedrich-Ludwig-Jahn-Straße 17, Greifswald, 17489 Germany

**Keywords:** Electron beam, Gamma irradiation, Medical plant safety, Ozone, Plasma-processed air, UV-C

## Abstract

The use of cannabis in medicine has rich historical roots spanning thousands of years before stigmatism surrounding the narcotic use of this plant led to strict regulations on its use. Recently, medicinal and recreational cannabis use has experienced a resurgence in many regions of the world, receiving attention from society and policy as legalization increases availability and necessity of greater mindfulness. Research on cannabis is gaining more traction to better inform the regulations needed to ensure the safe and effective usage of this drug. Owing to cannabis being a natural plant product, there are contamination risks even when it is produced under good manufacturing practices (GMPs), which can pose a health risk to consumers. Several pathogenic bacteria and fungi have been found on cannabis, either causing disease in the plant or causing health issues in people who consume the product. The literature on the microbiological safety of cannabis focuses on identifying the species that exist on the plant and the efficacy of sanitation methods currently used in the food industry for the decontamination of cannabis. Specifically, irradiation methods such as gamma irradiation and electron beams have been applied to effectively lower the microbial load on cannabis. However, these irradiation methods face pushbacks due to cost barriers, regulatory issues, and customer disapproval for irradiated products. Innovative gaseous and non-thermal plasma methods are beginning to gain attention because of their preliminary results in effectively decontaminating food products. Indirect non-thermal plasma methods such as ozone and plasma-processed air present new, possibly better, sanitation options for the complex flower structure of cannabis while maintaining its quality. This review discusses the historical importance of cannabis usage and the need to mitigate microbial contamination while considering the current methods used to sanitize cannabis. Finally, we present innovative gaseous and non-thermal plasma methods that are currently being integrated or are under research for cannabis sanitization to improve the safety and future regulatory aspects of cannabis processing.

## Introduction

The therapeutic application of cannabis in medicine has various historical roots, dating back to approximately 2,700 BC in China, where it was known as “ta ma” or "great hemp" (Chaachouaya et al. 2023). Cannabis was valued not only as a source of textiles, oil, and paper but also for its medical properties (Russo 2007). In the 19th and early twentieth century the use of cannabis as an active ingredient in form of tinctures and extracts was widespread in Europe and the United States of America (O'Shaughnessy 1843; Gieringer 1999; Sasman 1938). Recreational use of cannabis also increased with the surge of medical use during this time. However, medical cannabis use was curtailed in Germany with its inclusion in Germany’s Narcotics law by 1929. Following in the 1930’s, various laws in the USA led to a more widespread decrease in medical cannabis use (Däumichen 2016; Connor et al. 2021; Musto 1972). The World Health Organization's (WHO) Single Convention on Narcotic Drugs in 1961 changed the legal status of cannabis to an illegal drug, bringing its medical use to an almost complete standstill worldwide (Mills 2016; Nations 1961; Verbraucherschutz 1971). In 2024, medicinal and recreational cannabis use was legalized in Germany (Konsumcannabisgesetz – KCanG), allowing adults to possess 25 g (dried weight) of cannabis in public spaces and 50 g of cannabis in their residence (Justiz 2024). This legislation was the final step in many previous social and political discussions, as developments have shown that, despite existing prohibitions, cannabis use has been on the rise, particularly among young people. The main reason for this legislation was to reduce black-market trade because cannabis from this source is often associated with increased health risk, as cannabinoid content is unknown and toxic additives, impurities, and synthetic cannabinoids may be present—factors that cannot be estimated by consumers. The law aims to contribute to improved health protection, strengthen cannabis-related education and prevention, curb the illegal market for cannabis, and enhance the protection of children and young people. To protect consumers, the quality of recreational cannabis must be controlled, and the distribution of contaminated substances must be prevented (Bundesregierung 2023).

Cannabis and cannabinoids are also used in food preparation for medical and recreational purposes. The market for orally consumed cannabis edibles has expanded since it began being more widely legalized for recreational use. The preparation of cannabis-infused foods requires targeted thermal processing to convert the bioactive compounds into their active forms. This process begins with decarboxylation, in which the tetrahydrocannabinolic acid (THCA) contained in the raw cannabis flowers is converted into tetrahydrocannabinol (THC) by thermal action. The decarboxylation of THCA to THC takes place efficiently at temperatures between 110 °C and 145 °C (Wang et al. 2016). The optimal decarboxylation time is 30–45 min, depending on the temperature (Wang et al. 2016). The activated cannabis can be processed in a butter melt to extract the lipophilic THC from the flowers. The resulting cannabis butter can then be used for many different baked goods. Further possibilities are the use of cannabis extracts, raw cannabis itself, or, more recently, pure crystals of cannabinoids that can be added to the edible product.

In several countries, edibles are subject to strict regulations regarding the maximum THC concentration. In Canada, edibles containing THC may be sold with a maximum THC content of 10 mg per individual package or total package (Regulations 2019). In 17 of the 50 U.S. states, there are also legal THC limits for edible cannabis products, such as gummy bears or chocolate, which is usually 10 mg THC per serving and 100 mg per product (Swinburne 2022). In Germany, on the other hand, these THC-containing foods are strictly prohibited under the Cannabis Consumption Act § 21 para. 1 no. 3 KCanG (as of 05/2025) (Bundesamt für 2025). One reason for the higher presence of edibles in the market in North America compared to Europe is the strict restriction and complete non-admission in Europe.

THC is not the only valuable ingredient of the cannabis flower. Cannabidiol (CBD) is another popular cannabinoid available in several forms, such as edibles, tinctures, capsules, topicals, and vapes (Li et al. 2021). CBD is not psychoactive but is used therapeutically for a variety of conditions, including anxiety, psychosis, pain, inflammation, and epilepsy (Parker et al., 2022). Further, cannabis seeds contain a high content of fatty oil (25–35%) (Özdemir et al. 2021). His oil has a nutritionally valuable fatty acid profile with a high proportion of unsaturated fatty acids such as linoleic and linolenic acids (Deferne and Pate 1996). The food industry also makes use of the fact that cannabis seeds have a relatively high protein content (approx. 20–25%), which includes all the amino acids essential for human nutrition (für Risikobewertung 2018). These nutritional benefits led to the emergence of a rich market for various cannabis seed products in Germany, all of which contain very small amounts of cannabinoids due to the use of industrial cannabis (industrial hemp) instead of medical cannabis (für Risikobewertung 2018). Regardless of if medical or industrial cannabis is used, microbial safety and cannabis quality are required to guarantee consumer safety and acceptance.

### Safety challenges associated with the use of cannabis

In medical, recreational, and nutritional applications, various factors determine the safety of consuming cannabis products. For example, these products should be purchased legally by consumers to ensure proper processing standards and legitimacy of the product. One pressing safety challenge regarding cannabis consumption in is the sanitation of cannabis flowers, particularly concerning the removal of harmful human and plant pathogens. Other contaminants, like pesticides, heavy metals, aflatoxins, foreign particles, and insects are also challenging. These need other preventive or reactive methods like sanitation to be reduced or avoided. However, preservation of flower quality under treatment and storage conditions needs to be considered for current and novel sanitation processes. Due to a lack of consistent laws and standards worldwide, current sanitation and safety monitoring of cannabis remains flawed, particularly for cannabis that is internationally traded. This is because the importer and exporter countries may have different acceptance thresholds for contamination, which could require double safety and quality checks. The flawed monitoring of cannabis may lead to the unnecessary waste of flowers as these thresholds are different and certain decontamination techniques may not be accepted in the receiving country. This underscores the need for more consistent and reliable sanitation techniques and safety monitoring of cannabis.

The known microbiological risks associated with the consumption of cannabis flowers for medical or recreational use are introduced in this review. Additionally, the safety and quality needs of cannabis flowers for medical and recreational purposes will be highlighted, and a small discussion about the challenges on the regulation side in Germany and the European Union (EU) will be included. Furthermore, current sanitation methods available and used on the market, including their advantages and disadvantages, are described. Innovative methods are beginning to enter cannabis sanitation. Non-thermal plasma (NTP) methods, which have recently been proven to be effective decontamination techniques in plant and food applications, are of particular interest to effectively and safely remove microbiological contaminants from cannabis. This review provides a thorough overview of sanitation options, their respective advantages and challenges, gaps in knowledge, and future research needs.

## Microbiological risks of cannabis

During cultivation (outdoor and indoor) and at every stage of production, cannabis flowers are exposed to different sources of biological contamination. Microorganisms can be introduced by the water used for rinsing, by employees during production, or by the ambient air when the harvested cannabis flowers are dried (Holmes et al. 2015). Even producers who grow their cannabis flowers indoors and implement high standards of hygiene in their operations are not immune to contamination (Punja et al. 2019).

Under good manufacturing practice (GMP) of medical products, the contamination of the initial material can be reduced only through suitable hygiene measures and employee training. Production of cannabis flowers in the recreational sector provides comparable risks concerning microorganisms; therefore, GMP standards are also needed in this processing area. Researcher G.R. Thomson III has provided some insight into the microbial community of recreational cannabis flowers available on the market, including human pathogens, in his paper (Thompson et al. 2017). The detected microorganisms included gram-negative bacteria (*Escherchia coli*, *Salmonella* sp., *Enterobacter* sp., *Acinetobacter baumannii*, *Klebsiella pneumoniae*, *Pseudomonas* sp., and *Stenotrophomonas maltophilia*), gram-positive bacteria such as *Bacillus* sp. and molds such as *Aspergillus* sp. and *Penicillium* sp.

### Bacteria in/on cannabis

Bacteria are unicellular, prokaryotic microorganisms that do not have a cell nucleus (Slonczewski and Foster 2012). The vast majority of bacteria are nonpathogenic and fulfill essential ecological functions (Hahn et al. 2008). They play a central role in biogeochemical cycles and in symbiotic relationships with humans, animals, and plants, for example, in digestion, immune modulation, or nitrogen fixation. Nevertheless, pathogenic bacterial species exist that can cause disease in humans under certain conditions (Fuchs 2022). Two different forms of pathogenicity must be distinguished. Obligate pathogenic microorganisms must be completely eliminated from the organism to be considered safe, as they are pathogenic under all circumstances. Facultative pathogenic microorganisms usually belong to the physiological microflora, for example, the skin or mucous membranes, and only develop pathogenic effects under certain predisposing conditions (Hahn et al. 2008). The ability to produce harmful endo- and exotoxins makes microorganisms like *Salmonella* or *Escherichia coli*, which have been found to exist on cannabis, harmful for consumers. These toxins can trigger cytotoxic, enterotoxic, or neurotoxic effects and immune responses with symptoms like fever, diarrhea, and vomiting (Michael et al., 2013).

Cannabis leaves and flowers have a microbiome consisting of bacteria and fungi that grow on the surface (epiphytes) and that grow within the tissues of the plant (endophytes) (McKernan et al. 2016). Epiphytes may establish on the plant surface from liquids or aerosols or through human contact, whereas endophytes are known to enter the tissues from the rhizosphere—the soil environment directly surrounding the root—through the root junctions and subsequently spread via the xylem (McKernan et al. 2016; Compant et al. 2010). Some of these symbiotic bacteria are nonpathogenic; however, several pathogenic species have been found to exist in/on cannabis.

Legalization of cannabis products worldwide in recent years has led to a drastic increase in research publications focused on the presence of bacteria, particularly in medicinal cannabis (Alzate et al. 2025). McKernan et al., (2016) performed metagenomic sequencing on bacteria cultured from cannabis plant material and detected the following harmful bacteria at proportions of more than 5% of the total detected bacteria: *Acinetobacter baumannii, Acinetobacter pittii, Escherichia coli, Propionibacterium acnes, Pseudomonas aeruginosa, Ralstonia pickettii, Salmonella enterica* and *Stenotrophomonas maltophilia* (McKernan et al., 2016). Many of these species are pathogenic and/or toxigenic. *A. baumanni* is a highly persistent gram-negative species that is noted for being resistant to last-resort antibiotics and can cause pneumonia and bloodstream infections*,* with a mortality rate of up to 35% (Antunes et al. 2014). *E. coli* is a toxicogenic gram-negative bacterium that may lead to a variety of illnesses upon infection*,* including diarrheal illnesses, intestinal illnesses, and extraintestinal illnesses such as pneumonia, meningitis, and bacteremia, among others (Mueller and Tainter 2023). P*. aeruginosa* is gram-negative and typically does not cause infection in healthy people but can cause pneumonia and bacteremia in people with underlying diseases and even life*-*threatening chronic lung infections in people with cystic fibrosis (Moore and Flaws 2011). *S. enterica* is a gram-negative foodborne pathogen known to be the third leading cause of death among diarrheal diseases and one of the top causative agents of foodborne illness (Mkangara 2023). *S. maltophilia* is also gram-negative and is known for causing pneumonia because of its colonization of the respiratory tract and blood-stream infections (Looney et al., 2009). These bacteria can pose a dangerous threat to human safety when consumed; therefore, efforts to identify and quantify their presence on medical and recreational cannabis are of interest.

Another bacterial threat to cannabis plants and human safety through consumption is the possibility of pathogenic bacterial spores enduring on the plant. Spores are a form of survival of many microorganisms, especially bacteria and fungi, which are formed under unfavorable environmental conditions. In their dormant state, bacterial spores are metabolically inactive and extremely resistant to heat, desiccation, radiation and chemicals. They can remain inactive for long periods of time and return to their active, vegetative form when conditions improve (Setlow 2006). The genus *Bacillus* consists of gram-positive, facultatively anaerobic rod bacteria that can form endospores. Some members of this genus, such as *Bacillus cereus* and *Bacillus anthracis*, are known pathogens of food poisoning, pneumonia and anthrax (Hahn et al., 2008). While there is no current research confirming the presence of pathogenic *Bacillus* species or their spores on cannabis, some nonpathogenic *Bacillus* species are used as plant growth-promoting bacteria (PGPB).

The inoculation of seeds or plants with environmentally safe strains of bacteria has emerged as a sustainable method for enhancing crop growth and resiliency (Kulkova et al., 2023). Nonpathogenic strains of *Bacillus cereus* used as PGPB stimulate the growth of various crops, including soybean, wheat, and pea plants (Kulkova et al., 2023), whereas *Bacillus velezensis* has been proven to substantially increase cannabis growth (Aunkam et al., 2024). In addition to enhancing plant growth, PGPB species, including *Azosporillum brasilense*, *Gluconobacter diazotrophicus*, *Herbaspirillum seropedicae*, *Burkholderia ambifaria, Pseudomonas putida, Comamonas testosteroni, Citrobacter freundii, and Enterobacter cloacae*, have been used in studies and in industry to increase cannabinoid production in cannabis plants (Pagnani et al., 2018; Conant, et al., 2017). GPBs are also beneficially used as antagonist competition against phytopathogens, such as *Bacillus*, which inhibits the fungal pathogen *Fusarium oxysporum* on cannabis, improving plant resiliency (Corredor-Perilla et al., 2023). These bacterial species are regarded as safe for agricultural use and may reduce the microbiological risks of more harmful species on cannabis; however, their use may increase the total aerobic microbial count that is a parameter relevant for quality control of cannabis.

### Fungi in/on cannabis

To date, approximately 150,000 species of fungi have been scientifically described, classified, and named worldwide (Hawksworth and Lücking 2017). Fungi are capable of forming spores with a structural and functional design geared toward reproduction and efficient colonization of new substrates (Hahn et al. 2008). Depending on the group of fungi, spores can be formed exogenously on special structures or endogenously in fruiting bodies (Moore et al. 2020). While not the primary function of fungal spores, they are extremely resistant and can survive under extreme conditions. For example, spores of *Aspergillus*, which are potentially pathogenic for humans, can even survive extreme conditions in space, where they are exposed to constant cosmic radiation (Cortesão 2020). Insufficient control of the drying process of cannabis flowers after harvesting is associated with the risk of subsequent infection. The remaining moisture in the flowers can stimulate the fungal spores present on the plant to germinate, which can lead to microbial infestation of the harvested flowers (Virsik-Köpp n.d.). Spores are ubiquitous in our ambient air and can be avoided only through extreme hygiene measures, which are not applicable to the cultivation of cannabis flowers (Patel et al. 2018).

Molds are widespread in nature and appear macroscopically in everyday life, especially in foods such as bread, cheese or fruit (Deferne and Pate 1996). Each fungal filament, called a hypha, grows primarily at the tip via expansion of the terminal cells. These hyphae typically grow on a surface and form tuft-like complexes, the entirety of which is known as mycelium, which can be observed with the naked eye (Deferne and Pate 1996). Within hyphae, fungal spores can form, which are called conidia. This asexual resistant form serves to spread the fungus into new habitats (Deferne and Pate 1996). Although these conidia are present in the air throughout the year, they can cause invasive aspergillosis (IA), allergic bronchopulmonary aspergillosis (ABPA), or hypersensitivity (Linares et al. 2023). These mold conidia (e.g., *Aspergillus*) can play a pathogenically relevant role in medicinal cannabis in particular. For example, invasive aspergillosis associated with marijuana smoking has already been described in the past in two cancer patients receiving chemotherapy (Gargani et al. 2011).

In the context of the cannabis plant, the following species are among the most important phytopathogenic molds (Wang et al. 2016):

- *Botrytis cinerea*
  A necrotrophic mold that causes blossom blight known as “bud rot”, particularly under conditions of high humidity. The pathogen usually penetrates the tissue via mechanical injuries or natural openings and leads to massive decomposition of cannabis inflorescences (Mahmoud et al. 2023). Managing disease caused by *B. cinerea* is difficult because many fungicides do not effectively control pathogenic outbreaks due to their resistance mechanisms (Williamson et al. 2007). Additionally, most fungicides which are allowed for vegetables and fruits (e.g., Teldor, Luna experience, or Cuproxin progress) are not allowed to be used on cannabis. Here, biological fungicides like Diatical (based on diatomaceous earth) and Ekisan (based on horsetail) are preferred, and chemical fungicides (like Lexor25) are limited (Maldonado-Reina et al., 2021). *B. cinerea* is a known allergen and can cause harsh reactions in humans who inhale it (Jurgensen and Madsen 2009).
- *Aspergillus* spp.
  Ubiquitous airborne pathogens can grow on a wide variety of substrates. Spores of this epiphytic fungus have been observed to stick to the surface of cannabis bracts covered in glandular trichomes (Gwinn 2023). Individual species, especially *A. flavus* and *A. fumigatus*, are potentially pathogenic and are known to produce mycotoxins such as aflatoxins. These toxins may remain after the fungi is killed, as small protein structures are more challenging to destroy. Therefore, cannabis flowers should not only be examined for mold growth but also for toxin concentrations before use. The exposure of cannabis flowers to these toxins can pose a significant health risk when they are inhaled by users, especially immunocompromised patients (Gwinn 2023). Specifically, pulmonary aspergillosis is a common form of infection caused by these fungi and has been reported in immunocompromised cannabis users with HIV, type 1 diabetes, and cancer (Sciences et al., 2017; Remington et al., 2015; Salam and Pozniak 2017). Aspergillosis is the most common fungal infection among cannabis users, accounting for 43% of infections in one study (Gwinn 2023).
- *Penicillium* spp.
  These pathogens, prevalent during storage, are the most frequently recovered toxigenic fungi and are typical representatives of the postharvest microbiome of cannabis (Gwinn 2023). Penicillium spores stick to the surface of glandular trichome heads and can be embedded in sticky resin. Trimming flowers, including the removal of leaves and branches, can lead to an increase in the incidence of *Penicillium*. This is thought to be caused by the creation of entry points through injury to the plant tissue or the dispersal of endophytes from the pith tissue (Punja et al. 2019). *Penicillium* spp. found on cannabis are capable of producing mycotoxins such as ochratoxin A (OTA), a potentially nephrotoxic and carcinogenic secondary metabolite (Gwinn 2023). OTA can cause kidney damage in humans when they are exposed to even microgram amounts of the toxin (Bui-Klimke and Wu 2015).
- *Fusarium* spp.
  These species have been reported to infect the inflorescences, roots, and stems of cannabis plants. *Fusarium* species on the roots of Cannabis cause browning and decay of the roots, resulting in yellowing and sometimes death of the plants (Punja et al. 2019). Additionally, *Fusarium* spp. have the potential to produce spores on stem tissues, which can spread to the air or water to further cause bud infection in surrounding plants (Punja et al. 2019). This also presents a safety risk for cannabis workers, as airborne spores may be inhaled. *Fusarium* commonly produces the vomitoxin deoxynivalenol (DON), which causes symptoms such as nausea, vomiting, diarrhea, and even death (Ji et al. 2019).

### Other microorganisms on cannabis

Fungi represent the greatest microbiological threat to plant and human health for cannabis, with bacteria also contributing to danger; however, other microorganisms, such as viruses, parasites, and archaea, may affect cannabis plants during cultivation. Since 1941, reports have increasingly revealed that viruses and viroids affect hemp and cannabis production worldwide (Chiginsky et al. 2021; Röder 1941). Recently, viruses such as cannabis cryptic virus (Righetti et al. 2018), hop latent viroid (Warren et al., 2019), lettuce chlorosis virus (Hadad et al. 2019) and beet curly top virus (Chiginsky, et al. 2021) have been shown to infect cannabis plants, leading to symptoms such as leaf wilting, malformation, brittleness, and yellowing.

Studies on cannabis with root rot reported that several *Phythium* spp., including *P. dissotocum* and *P. myriotylum,* were isolated from symptomatic tissue, yielding more severely rotted roots than those caused by *Fusarium* fungi (Punja and Rodriguez 2018). *Pythium* spp. on cannabis are pathogenic oomycetes that are fungus-like organisms but are separated from true fungi (Chenari Bouket et al. 2013). Nematode parasites have also been shown to invade cannabis plants, leading to stunted growth, wilting, and reduced crop yield (McPartland 1996). Five root-knot nematode species from the *Meloidogyne* genus, with *M. incognito* being the most common, have been shown to create galls in the roots of hemp plants and have been associated with stunting (Bernard et al. 2022; Thiessen et al. 2020). Lesion *Pratylenchus* nematode species also reproduce on cannabis and are often associated with wilt fungi on other plants such as cotton, creating destructive disease complexes; however, no research has revealed these disease complexes on cannabis (McPartland 1996; Núñez-Rodríguez et al. 2023).

Pesticides are commonly used in agriculture to reduce the number of microorganisms, such as the presence of nematodes or fungi, on plant products; however, the use of various pesticides can have lingering effects on cannabis. Fungicide treatment of cannabis has been shown to affect the diversity of the rhizosphere microbiota and increase the abundance of Archaea in the plant rhizosphere (Xu et al. 2024). Additionally, it has been determined that nematicides need to be avoided in recreational and medicinal cannabis production (Bernard et al. 2022). Pesticide residues have been found to reside on cannabis samples, particularly purchased from illegal sources but also have been found on some legal samples, which can be dangerous for the consumer (Russo 2016). Human consumption of the chemicals used in pesticides can cause an array of health disorders, and these chemicals can be transferred from cannabis to the user through methods such as smoking, eating, or vaporizing, for example (Dryburgh et al. 2018).

## Regulatory, quality, and safety requirements for cannabis

### Regulatory requirements

Like other medicinal products, cannabis flowers are subject to regulatory requirements. To produce medicinal cannabis in Germany and the EU, manufacturing authorization in accordance with GMP guidelines must be obtained. These manufacturing licenses are issued by the respective states or federal states. The cultivation of cannabis flowers must also be strictly monitored by growers. Some producers of cannabis flowers choose to grow their flowers under GMP conditions (DEMECAN 2022). However, other producers initially cultivate under the controlled but looser standards of the Good Agricultural and Collection Practice (GACP) and only refine the phytopharmaceutical under GMP conditions during further processing of the plant into a derived product such as an oil or extract (Cantourage 2024). If cultivation is not carried out directly by the producer of the medicinal product, the latter is obliged under GMP regulations to continuously monitor its growers with regard to their cultivation.

Regardless of the transition from GACP to GMP, producers must adhere to the strict requirements for medicinal cannabis. For every variety that is available in Germany, a prior registration and application for a permit in accordance with § 4 of the Medicinal Cannabis Act (MedCanG) must be submitted (Bundesministerium n.d.). This application must be made individually for each chemovar and for different THC concentrations with a range of ± 10% based on the THC target value. The flowers must also be registered in the same way with all companies involved in the supply chain to ensure seamless monitoring of the medicinal product. Only then can the flowers be processed into a medicinal product in Germany and distributed to pharmacies. The legal basis for CBD products in Germany and the EU is complex. On the one hand, CBD is subject to prescription in Germany as a medicinal product (Bundesministerium n.d.). On the other hand, the same compound is also available as a CBD-containing oil in pharmacies with 2–25% CBD content without a prescription as an over-the-counter dietary supplement (Boyar 2021). Other products, such as creams containing CBD, are considered cosmetics, and some CBD-containing mouth sprays are classified as food (Solmecke 2024).

### Quality requirements

The domestication of narcotic *Cannabis sativa* varieties involved the targeted selection of floral color characteristics, such as purple or white strains (Small 2015). These visual characteristics are considered indicators of high potency and are therefore preferred for further breeding. The popularity of such color characteristics clearly shows the influence of color trends and human aesthetics on cannabis breeding goals, which is also reflected in consumers' purchasing decisions. The visual assessment of color and its association with the perceived quality of cannabis flowers is widespread in the recreational consumption market and largely agreed upon among consumers. Bright, shiny green tones are generally considered indicators of high-quality flowers, whereas dark green, brownish, or pale shades are often associated with inferior quality (Hartfield 2024). The texture of cannabis also plays a decisive role in the assessment of quality for many consumers. Ideally, cannabis flowers should be slightly sticky and spring back when pressed. These properties are mostly related to the residual moisture content of cannabis flowers.

Cannabis evaluation, especially for the recreational use, is primarily based on trichome density, coloration, the intensity and complexity of the odor profile, texture, and patient safety (Rothmeier 2025). For pharmaceutical quality assessment, the focus is primarily on patient safety. The European Pharmacopoeia primarily examines safety-relevant aspects such as the content of the herbal drug, pesticides, heavy metals, microbiological contaminants, and mold toxins (EDQM 2023).

Perhaps the greatest interest is in the compounds that lead to the “entourage effect”, which is the positive effect of terpenes on the active cannabinoids (Ferber et al. 2020). Testing the terpenes responsible for the odor and entourage effect is not required according to the European Pharmacopoeia monograph (EDQM 2023). However, the Food and Drug Administration (FDA) has stated that terpenes are important for the identification of cannabis cultivars because of their specific odor and flavor and to ensure the strength of the final product (Pruyn et al. 2022). While there are over 100 cannabinoids in *Cannabis sativa*, quality requirements typically focus on tetrehydrocannabinol (THC), cannabidiol (CBD), tetrahydrocannabinolic acid (THCA), and cannabidiolic acid (CBDA) (Pruyn et al. 2022). Cannabinoid content varies based on factors such as the chemotype, growth, and storage conditions of the cannabis (Sarma et al. 2020). Therefore, cannabinoid requirements typically state that medical cannabis products must contain only cannabinoids from the cannabis plant, not synthetic cannabinoids, and that the chemical structure must not be modified or transformed (Austrailian 2017; Moody et al. 1982).

### Safety requirements

Pathogenic fungi and bacteria on cannabis can pose a particularly high risk to immunosuppressed patients. Additionally, the argument that inhaled smoke (50–60°C) is sterilized has been proven false, as some microorganisms present on cannabis are able to recolonize in cultivation media even after water filtration from smoking cannabis through a water pipe (Moody et al. 1982). Therefore, special care must be taken with medicinal cannabis flowers that have not undergone any germ-reducing treatment. This is because pathogenic germs such as *Escherichia coli*, *Pseudomonas aeruginosa*, *Bacillus cereus*, or *Aspergillus* spp. can colonize the pipes of growers' irrigation systems and cause spot contamination on cannabis plants that may not be detected during random testing. To minimize this risk, cannabis flowers intended for medicinal purposes should always undergo germicidal treatment.

Although medicinal cannabis flowers offer a certain degree of safety for patients because of their direct control and regulated production conditions, even pharmaceutically produced batches are only subject to random analysis. This is because microbiological testing according to the European Pharmacopoeia (Ph. Eur. 2.6.31) is a destructive test method, which makes a complete examination of each individual flower impossible (Medicines 2020). According to monograph 5.1.8 “Microbiological Quality of Herbal Medicinal Products for Oral Use” of the European Pharmacopoeia, the microbiological quality control of herbal medicinal drugs, such as cannabis flowers, is carried out by determining the total microbial count, which is the sum of the total aerobic microbial count (TAMC) and the total yeast and mold count (TYMC), among other factors (Medicines 2023).

Across the world, the rules for the acceptable limits of TAMC and TYMC on medical and/or recreational cannabis vary (Table 1). Depending on the intended area of application, many countries have regulations for the complete absence of some microorganisms, such as bile salt-tolerant gram-negative bacteria, *Escherichia coli, Salmonella*, *Staphylococcus aureus*, and *Pseudomonas aeruginosa* (CARO Analytic Services 2021; Austrailian Government Theapeutic Goods Administration 2020).

**Table 1:** Acceptable limits of TAMC and TYMC on cannabis flowers for different countries

Country/union	TAMC (CFU/g*)	TYMC (CFU/g)	reference
USA	10^2^–10^5^	10^2^–10^5^	(Genomics 2025)
EU	10^3^ 10^2^ (for inhalation)	10^2^ 10^1^ (for inhalation)	(Medicines 2023)
Canada	10^5^	10^4^	(CARO Analytic Services 2021)
Australia	10^2^ (for inhalation)	10^1^ (for inhalation)	(Austrailian Government Theapeutic Goods Administration 2020)

In addition to microbiological safety, there are regulations concerning the presence of heavy metals, pesticides, and foreign matter contamination on cannabis (Pruyn et al. 2022); however, germ reduction is the focus of this manuscript. As long as there are no uniform standards for recreational cannabis that meet the requirements of a medicinal product, it is crucial for patients to strictly separate medical cannabis and cannabis for recreational use. However, complete elimination of bacteria and fungi from cannabis flowers is unrealistic. Some cannabis-producing companies are reluctant to use germ reduction processes, as these processes are sometimes associated with high costs, bureaucratic effort, and potential losses in organoleptic quality. Therefore, the development of a cost-effective, license-free, and quality-preserving germ reduction process represents a significant step toward improving the safety and efficacy of both medicinal and recreational cannabis use.

## Current applicable irradiation methods for cannabis sanitation

### Gamma irradiation

Gamma irradiation is an established physical process for the decontamination of cannabis flowers. In particular, in Canada and the Netherlands, it is often used to reduce the germination of cannabis flowers to comply with low microbiological requirements (Cannabis 2026). Using high-energy gamma photons, which are typically generated by a Cobalt-60 source (Haji-Saeid et al., 2007), the process of gamma irradiation uses ionizing radiation to damage the DNA of microorganisms, leading to the inactivation of bacteria, fungi, pests, and even spores (Virsik-Köpp n.d.).

The radiation dose in which the cannabis flowers are to be exposed to must be determined individually by the respective flower producers. For irradiation, the dose is dependent on initial contamination levels, the packaging unit, and the orientation of the flowers and must be validated in advance before it can be used regularly for the GMP-compliant production of medicinal products. Before the irradiation efficacy can be tested, the natural microbial load (bioburden) on the product must be tested first. However, unlike medical devices, which are manufactured under standardized conditions, the bioburden of cannabis flowers is not reproducible from batch to batch because they are a natural product. Different values regarding the radiation dose currently used for cannabis in industry are mentioned in the literature, and various factors influence the selection of the required radiation dose. In Canada, most cannabis flowers are regularly treated with gamma radiation at doses of approximately 15–20 kilograys (kGy) (Majumdar et al., 2023). However, an Israeli study that tested gamma and e-beam radiation against a cold plasma vacuum also revealed sterilizing effects on certain germs already at doses of 7.5–8.37 kGy (Jerushalmi et al., 2020). In addition, studies have shown that bacterial growth can be inhibited at a radiation dose of 2.5 kGy (Frink et al., 2022). A study in the Mississippi showed that a dose of 15–20 kGy allows for deep penetration into the plant tissue and thus enables sufficient sterilization to generate even lower limit values of < 10 colony-forming units (CFUs) (Majumdar et al., 2023).

Gamma irradiation of other products is a well-researched field and is used in other industries, such as the food industry, which might provide more insight into its application for cannabis. For example, in 1999, a study group appointed by the WHO concluded that food treated with a radiation dose is both safe to eat and nutritionally harmless (WHO 1999). However, the irradiation of cannabis flowers also has several disadvantages. For example, gamma irradiation has been found to have a negative effect on the terpene content of myrcene and linalool in fresh coriander or other terpenes, and a similar effect in cannabis can have a synergistic effect on the bioavailability of cannabinoids (Russo 2011; Fan and Sokorai 2002). In a Dutch study on the well-known cannabis product Bedrocan®, which was supported by the Dutch Ministry of Health, a radiation dose of 10 kGy did not affect the contents of THC and CBD but did lead to a decrease in some other terpenes that were not determined to be significant (Hazekamp 2016).

While gamma irradiation seems to be an effective method for the decontamination of cannabis with little documented effect on quality, this process faces barriers for acceptance due to ionizing radiation licensing, cost and time, and customer approval. For example, in Germany, cannabis products treated with ionizing radiation require an AmRadV license for each strain that is treated (Bundesinstitut für Arzneimittel und Medizinprodukte 2022). This licensure application costs €4500 per strain and can take a full year to be approved, representing a large cost and time barrier (Ziel 2024). Additionally, gamma irradiation itself is expensive, as it requires radioactive isotopes (which are also not environmentally friendly), specialized facilities (proper shielding of the radioactive source for operator safety), and costly equipment (Silindir Gunay and Ozer 2009; Lastname et al. 2020). Finally, irradiated cannabis drives down customer approval and might even lead to consumers choosing nonregulated cannabis products. In Canada, irradiated edible cannabis must be labeled with the international symbol, the Radura, and a statement that the product has been treated with ionizing radiation (Government n.d.). Due to this labeling, the cannabis cannot be considered organic, which has recently led to trends in which consumers steer clear of this product (Ziel 2026). In the United States, there is also skepticism regarding irradiated cannabis, as consumers have complained about low-potency and flavorless flowers in Michigan, and there is a fight for the Radura label to be mandatory in Nevada to ensure transparency in processing and cannabis quality (Steele 2021; Willow 2021).

### Electron-beam (e-beam) sterilization

Electron beam (e-beam) sterilization is another physical process used for the decontamination and sterilization of materials via high-energy electron radiation. In this process, accelerated electrons are used to generate ionizing radiation. Like gamma radiation, direct DNA damage is caused in microorganisms, preventing them from replicating, including spores (Silindir Gunay and Ozer 2009). Compared with gamma irradiation, e-beam technology has a lower penetration depth because electrons interact extensively with a material as they pass through it, causing them to lose energy (Naikwadi et al., 2022). However, this makes it possible to achieve more precise dose control and shorter irradiation times. E-beam treatment can achieve comparable microbial reduction rates at very short exposure times (approximately 15 min compared with 24 h with gamma radiation) (Silindir and Özer 2009). The greatest advantage of this method is that no radioactive isotopes, such as Cobalt-60, are needed; therefore, no major harmful substance in the form of radioactive waste is caused by the method (AST 2026).

An e-beam dose of approximately 10 kGy was sufficient to completely decontaminate cannabis flowers, with an initial microbial load of approximately 10⁸ CFU/g (Jerushalmi et al. 2020). Higher doses of electron radiation (150–450 kGy) can induce structural changes in the cell components of *Cannabis sativa*. It has been shown that treatment with electron beams leads to a significant reduction in xylan, which promotes structural breakdown of the plant cell wall and significantly improves the enzymatic hydrolysis processes (Sung and Shin, 2011). Structural changes in cell architecture were also suspected at lower doses of electron irradiation (5 kGy). In a study on the effect of e-beam radiation on cannabis flowers, a significant increase in the measured cannabinoid content was observed in 4 different samples. The authors attributed this finding to radiation-induced changes in the cell structure, which may facilitate the release and thus the extractability of THC in the solvent, thereby explaining the observed increased concentrations (Kovalchuk et al. 2020). The terpene content in cannabis has been reported to decrease, particularly in the period immediately after e-beam treatment (Goffman et al. 2025).

As previously mentioned for gamma irradiation, e-beam treatment faces similar regulatory barriers due to the use of ionizing radiation to decontaminate cannabis to acceptable levels. The e-beam process is considered to be less expensive than gamma irradiation because it does not require radioactive isotope supplies and therefore, complex safety-process management costs and the source shielding; however, the construction of facilities with proper e-beam equipment can be expensive (Silindir Gunay and Ozer 2009). Additionally, changes in the terpene and cannabinoid contents may lead to decreased customer approval and loss in sales, which in turn could challenge the cost-effectiveness of this sterilization option.

### Ultraviolet C (UV-C) radiation

For this method, microbial inactivation is achieved by means of the electromagnetic spectrum, which has a particularly effective antimicrobial ability at wavelengths between 200–280 nm (nm). This range is referred to as UV-C (Rahmati et al. 2022). While UV-B radiation (280–320 nm) has a lower inactivation effect, UV-A radiation (320–400 nm) has a neutral to beneficial effect on microorganisms. UV-C is created using a source that can obtain a high temperature, which is typically a plasma source.

UV-C radiation has a strong genotoxic effect on all types of microorganisms, such as bacteria, fungi, and viruses (Vanhaelewyn et al. 2020). UV rays penetrate the cell membrane and damage the DNA and RNA of microorganisms, preventing them from reproducing and causing cell death. UV-C radiation is used in many applications, including food processing and surface decontamination. Both of these applications can inform the further usage of UV-C technology for cannabis safety. Regardless of the application, the extent of microbial inactivation is directly proportional to the dose of UV-C received, relies on the type of microorganism, and is based on the intensity, distance, and duration of decontamination (Fan et al. 2017; Górny et al. 2024).

UV-C has been beneficially used in food processing to reduce the microbiological load from the surface of fresh produce, among other food products (Fan et al., 2017). For example, when UV-C light with a wavelength of 254 nm was applied to spices such as hot pepper, fennel, and coriander, a significant reduction in the viable count of bacteria and total fungi (CFU/g), including *Salmonella* spp. and *E. coli*, was observed (Hassan et al., 2020). An effective application involving a reduction in the microbial load without changes in color or sensory properties was carried out on dried bay leaves and oregano, among others. These products can be compared with medicinal cannabis on the basis of their properties and indicate the effectiveness of treating medicinal cannabis with UV radiation (Rahmati et al., 2022). However, one pitfall of UV-C decontamination of foods is that there can be a shadowing effect due to three-dimensional shape of the food, which limits its penetrating power and can prevent some microorganisms from being inactivated (Fan et al., 2017). This is particularly relevant for cannabis flowers, which do not have a smooth surface but, due to their natural flower structure, have a particularly large and uneven surface, which could limit the use of UV decontamination for cannabis flowers.

UV-C has proven to very effectively remove pathogens from plastic, metal, and glass surfaces, including *Pseudomonas aeruginosa* and *Aspergillus versicolor,* which are relevant contaminants of cannabis flowers, as previously mentioned (Górny et al., 2024). Cannabis flower products are commonly packaged in plastic or glass containers by distributors (Saputo n.d.). The ability of UV-C to decontaminate these surfaces points to the possibility that UV-C can be used on packaging material for cannabis to further ensure microbiological safety when the product reaches the consumer.

Legislation varies worldwide for the use of UV-C for food decontamination. UV-C has been approved in the United States by the FDA under regulation 21CFR179.39 for safe surface decontamination of foods (U.S. 2005). Regulations for UV-C treatment under the European Union (Regulation (EU) 2015/2283) and Canada focus primarily on UV-treated foods being considered as novel foods because of possible changes in nutritional content (European 2017; Government 2024). Additionally, many regulations focus on the application of UV-C in liquid foods. While these regulations may inform the necessary guidelines and rules for UV-C decontamination of cannabis, there is currently a need for this technology to be tested on cannabis to understand if it is compatible for efficient decontamination before regulations can be formulated.

## Current applicable gaseous sanitation agents for cannabis

### Ozone

Ozone is a colorless and reactive gas that is denser than air in its gaseous form (Jadhav et al. 2021). In an ozone generator, oxygen molecules (O_2_) split into free oxygen atoms via energy induced bond breaking, react with each other, and form ozone molecules (O_3_) (Rahmati et al. 2022). Ozone generators usually employ plasma, such as a dielectric barrier discharge (DBD), by the action of UV radiation or electrical discharge for the high energy needed to form ozone (Siemens 1889). Due to the instability of ozone compared with atmospheric oxygen, it must be continuously generated on demand via an ozone generator (Epelle et al. 2023). The ozone is then injected into a chamber, either in a gaseous or aqueous phase, for decontamination of various surfaces, medical equipment, water, and food (Epelle et al. 2023).

Ozone decontamination has been used in food processing for decades throughout many countries and has gained the status of a generally recognized as safe (GRAS) antimicrobial agent in the United States (Oner et al., 2016). Treatment with ozone is associated with the possible oxidation of double-bonded cellular components, damage to cell membranes, altering their permeability, damage to protein structure leading to enzyme malfunction, and ultimately, cell death (Rahmati et al., 2022; Jadhav et al., 2021). This method is effective for a wide range of microorganisms because of the high oxidative power and rapid decomposition of ozone (Epelle et al., 2023). Ozone treatment is typically used for surface decontamination of food products but also has the benefit of possibly removing residues of mycotoxins and pesticides from the surface of agricultural products (Kim et al., 1999). Factors that influence the decontamination effect of ozone technology include the type of microorganisms, amount of microbial contamination, temperature, pH value, and relative humidity (Epelle et al., 2023).

Ozone has been successfully used as a decontamination method for food products with properties similar to those of medicinal cannabis. For example, microbial colonization was reduced in treated herbs such as thyme, tea, and dried oregano (Rahmati et al., 2022). Additionally, ozone treatment has been proven to control *Fusarium verticillioides*, *Penicillium* spp., and *Aspergillus flavus*, which are relevant fungal pathogens on cannabis, in maize (Epelle et al., 2023). The cannabis industry has already taken an interest in the use of this method as an effective way to reduce the total yeast and mold count. There is a Canadian patent for a device designed specifically for ozone decontamination of cannabis (Willowpure 2024). The system consists of a closed ozone chamber with special racks or drawers for cannabis plant material. An oxygen concentrator generates ozone from ambient air, which is fed into the chamber via an ozone regulator. An integrated control system continuously measures the ozone concentration, compares it with a specified target value, and adjusts the gas flow accordingly. The target concentration is usually set between 200 and 400 ppm, with a treatment time ranging from 20 min to 48 h.

The use of ozone for decontamination of cannabis stands out as a beneficial method for a few reasons. First, this method uses gas contact for decontamination instead of a light, plasma, or radiation source to directly contact the microorganism. This is a great advantage for cannabis because of the complex structure of cannabis flowers, with many spaces between the calyx, leaves, and trichomes, in which shadowing effects prevent the other methods from effectively reaching those hidden surfaces. Second, ozone’s status as GRAS and comprehensive usage in the food industry may help streamline the process of implementing regulations for cannabis treatment so that this method can be commercially available soon. Finally, compared with chemical disinfection methods, ozone is considered to be cost effective and environmentally friendly because it decomposes back into oxygen gas and does not create any toxic byproducts or residues (Absolute n.d.). Therefore, it represents a safe and affordable option for decontaminating cannabis that does not require chemicals or irradiation, which could lead to customer disapproval.

However, while ozone is safe for the products being treated, protective measures need to be taken for the workers performing the treatment because ozone inhalation can cause severe lung damage and irritation of the respiratory tract (Epelle et al.. 2023). This may require additional costs to ensure proper protective equipment and training to keep personnel safe as ozone application offers in-house application possibilities without the security of an external professional company. It is also currently inconclusive whether ozone treatment may cause adverse quality changes, such as in color, odor, or texture, of other food products or medicinal plants and, in some cases, has even been shown to improve these qualities (Epelle et al.. 2023; Shafiee and Hosseinzadeh Samani 2024). Due to the highly oxidizing nature of ozone, cannabis plant material might be altered if treated incorrectly, which could lead to massive product losses. Further research on the effects of ozone treatment, specifically on the quality of cannabis, is needed to optimize the treatment parameters to prevent adverse effects while still achieving high microbial reduction and to determine whether this method would affect consumer approval.

### Vacuum-Water Vapor-Vacuum (VWV) process

The VWV is a process for germ reduction based on the principles of pasteurization and the thermal destruction of microorganisms (Vegara et al. 2013), excluding spores and thermophilic bacteria. Pasteurization was initially used only for liquids and is widely used in the dairy industry (Holsinger et al., 1997). The process used for vegetables, herbs, spices, mushrooms, teas, and pharmaceutical raw materials such as cannabis flowers is a further development of this original process. VWV involves a cycle of evacuations and saturated steam exposure that are carried out in an autoclave chamber. An important aspect of this process is the absence of any chemicals, and only the combination of heat, water and vacuum contributes to germ reduction. Therefore, VWV is also approved for organically produced products. The aim of this process is to achieve a reduction in bacteria by one to four orders of magnitude, with the sensory properties of the products being affected as little as possible. This process may be effectively used to sanitize cannabis products without significantly changing the organic properties of the plant; however, public research is needed to understand its application in this field as current research is only available for companies with patents for this technology.

## Innovative non-thermal plasma sanitation methods

As mentioned above, the current methods for decontaminating medicinal cannabis have several disadvantages, such as regulatory issues, cost barriers, and decreased customer approval. To counteract these disadvantages, new non-thermal plasma methods are being researched. Some of these plasma methods have already been used to decontaminate medicinal and aromatic plants which provides a scientific basis for their use in medicinal cannabis (Rahmati et al. 2022).

Plasma is generally regarded as the fourth state of matter. Plasma can be generated under a wide variety of operational conditions, as evidenced by naturally occurring plasmas such as lightning, the sun and stars, or the intergalactic nebula (Hu et al. 2024). In technical plasmas, plasma is defined as a partially ionized gas typically produced by electrical breakdown of a gas (Erich and Kunhardt 2013; Hale 1948). Through this ionization, free charge carriers, such as electrons and ions, are available to conduct an electrical current. This electrical conductivity enables the use of different types of electrical power supplies to sustain this plasma state.

It is important to distinguish between thermal and non-thermal plasmas. While thermal plasma is in thermodynamic equilibrium, which is achieved typically at temperatures of several thousand K, non-thermal plasma is characterized by a thermodynamic imbalance caused by energetic electrons colliding with heavier ions and neutral species. This energy transfer occurs mainly to the neutral molecules, whereas the bulk gas remains at or near ambient temperatures (Niemira 2012). For the purpose of this review, we restrict on the use of non-thermal atmospheric pressure plasmas and air as the process gas.

These low temperatures are crucial for the decontamination of microorganisms in temperature-sensitive materials, food, and potentially cannabis (Niedźwiedź et al., 2019). Plasmas work effectively against microorganisms because of the creation of reactive oxygen and nitrogen species. These species interact with amino acids in proteins, causing structural changes and damage to the cell in addition to directly damaging nucleic acids (Cherif et al., 2023). This approach enables the inactivation of vegetative bacteria, bacterial spores, yeasts, and molds (Table 2) under non-thermal conditions using energetic and reactive gases (Hertwig et al. 2015a). Various NTP methods have been shown to be relevant in the processing of cannabis. For example, non-thermal plasma treatment with dry air had strong fungicidal effects on *Aspergillus* spp. spores found on hazelnuts after only 5 min of treatment (Dasan et al., 2017). *Aspergillus* spp. are among the most dangerous pathogens for consumers of cannabis, and this fungicidal effect of NTP would be beneficial. Additionally, plasma jets have been shown to increase the drying efficiency of cannabis when used as a pretreatment, which in turn reduces the risk of microbial growth while retaining the terpene and cannabinoid contents of cannabis (Das et al., 2025).

**Table 2:** Mechanisms of non-thermal plasma action on individual microorganisms

	**bacteria**	**bacterial spores**	**yeasts**	**molds**
Inactivation mechanisms of cold plasma	1. destruction of the cell membrane 2. DNA damage 3. inactivation of proteins	1. oxidation of the outer membrane by a combination of reactive oxygen species (ROS) + dipicolinic acid release	1. oxidative processes 2. inactivation of enzymes	1. destruction of the cell wall 2. lipid oxidation leading to necrosis
Special features	1. gram-negative bacteria are more sensitive than gram-positive bacteria due to thinner cell wall 2. generally high effectiveness in bacteria due to many inactivation mechanisms	1. increased effectiveness of plasma treatment due to dry conditions	1. increased plasma effect due to water as a medium 2. effectiveness of plasma treatment depends on the gas composition and the density of the yeast cells	1.increased effectiveness due to long treatment times and increased performance of plasma treatment

In Israel, a non-thermal plasma system called SteriGreen™ was developed by the NovaGreen Company (Kibbutz Megiddo, Israel) specifically for the treatment of cannabis inflorescences (NovaGreen n.d.). This system uses a radio frequency (RF) generator to produce the plasma and short cycles of low-temperature hydrogen peroxide vaporization. NTP treatment of cannabis using this system revealed efficient fungicidal effects on uninoculated inflorescences (3 log CFU/g reduction after 10 min of treatment) and antibiotic properties on inoculated inflorescences with *B. cinerea* (4 log CFU/g reduction after 10 min of treatment) (Jerushalmi et al., 2020). While a system such as this proves the possibility of non-thermal plasma being used for cannabis sanitation, it relies on hydrogen peroxide to generate the reactive plasma and resulting oxygen species. This may increase customer disapproval due to the use of a chemical agent, although it does not directly contact cannabis and additionally it increases supply costs for the processor. Further research on NTP methods that do not require the use of a costly chemical or noble gas to achieve effective cannabis sanitization could enable the development of a practical system that can be implemented in the cannabis industry.

### Plasma-Processed Air (PPA)

Plasma-processed air is produced by energized interactions between neutral- and free-charged particles with air molecules in a closed space creating, primarily, reactive nitrogen species (RNS) (Schnabel et al., 2021). The composition of the plasma is influenced by factors such as the plasma source and working gas; however, the active species within the PPA from a microwave source mainly consist of N_x_O_y_ RNS, such as nitrogen oxide (NO) and nitrogen dioxide (NO_2_) (Winter et al., 2023). In the case of PPA, plasma generated via microwaves or DBDs and compressed air has been shown to exhibit antimicrobial abilities across various applications, including the decontamination of food (Winter et al., 2023; García Casado et al., 2024), seeds (Wannicke et al., 2021), plants (Schnabel et al., 2018), and other surfaces (Kramer et al., 2020; Katsigiannis et al., 2022; Schnabel et al., 2014).

The reactive species in PPA have powerful antimicrobial effects because of their ability to oxidize nucleic acids, lipids, and proteins (Domonkos et al., 2021). One proposed mechanism of destruction is that the reactive species first accumulate on the microorganism surface, diffuse through the cell membrane, and then disrupt the homeostasis of the intracellular pH, causing the inactivation of bacterial cells (Hertwig et al., 2015b). In particular, microwave-generated PPA has been successfully applied to inactivate enzymes (Bußler et al., 2017), pathogenic bacteria (Baier et al., 2013), and bacterial spores (Hertwig et al., 2015b) in various fresh and dry foods. However, the effect of PPA treatment on the quality of different foods varies based on the food type being treated. For example, PPA treatment did not affect the color of yellow peaches (Wu et al., 2022) and prevented blackening of potato tissue (Bußler et al., 2017) but led to browning of carrots and negatively impacted the physiological activity of cucumber tissue (Baier et al., 2015). These adverse quality impacts also corresponded to the treatment parameters that were most effective at reducing microbial contamination of carrots and cucumbers. Nevertheless, the results of PPA treatment of food products highlight the need for specialized treatment parameters for each individual product to effectively remove microorganisms while preserving quality.

PPA treatment of dry foods is of particular interest with respect to quality retention and decontamination because it may provide a basis for how this method could be used to treat cannabis due to its dryness (with an ideal a_w_ of 0.55 to 0.65). A study by Hertwig et al. explored the inactivation of the native microbial flora on whole black pepper seeds, red paprika powder, and crushed oregano by PPA (Hertwig et al., 2015a). Treatment for 5 min inactivated all molds and 30 min significantly reduced the total mesophilic aerobic count of black pepper seeds; 60 min inactivated molds and 30 min reduced the total mesophilic aerobic count below the detection limit for crushed oregano; and 60 min resulted in the highest inactivation of the total mesophilic aerobic count for red paprika powder. The authors of Casado et al. add on to this by showing that PPA treatment significantly reduced the natural total mesophilic aerobic count of wheat grains after 30 min of treatment, but PPA had no significant antimicrobial effects on onion flakes inoculated with bacteria (García Casado et al., 2024). Both of these studies reported that the inactivation of microorganisms depends on the composition of the microbial load and the surface structure of the dry product being tested. Cannabis has its own unique surface structure, including increased complexity of flowers and native flora; however, the treatment times and parameters used for other dry foods could provide a basis for introducing PPA treatment for cannabis.

Additionally, the quality impacts observed in the study from Hertwig et al. could provide insight into the potential effects on cannabis quality, namely color. PPA treatment caused color changes in oregano, possibly due to the destruction of chlorophyll, and significant changes in red pepper powder to a lighter, yellow color were proposed to be linked to the oxidation of carotenoids (Hertwig et al., 2015a). These findings suggest that PPA more strongly affects carotenoid-related coloration than chlorophyll-related coloration, a conclusion further supported by Baier et al. (Baier et al., 2015). These pigments are key contributors to the coloration of cannabis and are already susceptible to changes during drying and storage of the plant. Subsequent significant color changes from PPA treatment should be avoided, if possible, to maintain customer approval and satisfaction, particularly for recreational users.

Plasma-processed air is not yet used commercially in the food industry; therefore, regulatory aspects and decontamination systems must still be piloted before this treatment can be widely implemented. Experiments on the application of PPA treatment to cannabis are underway to determine the method parameters that could be used commercially to decontaminate cannabis. A potential system is currently being tested in house at the Leibniz Institute for Plasma Science and Technology (data not yet published) by the authors of this review paper. The system includes a microwave plasma source that generates PPA from compressed air, which is cooled in transit through tubing before entering a chamber with racks of cannabis flowers. Ideally, cannabis will be exposed to the PPA for a duration long enough to diffuse into the hard-to-reach surfaces of the flowers for proper decontamination without significantly changing the quality (e.g., color, texture, cannabinoid profile, relative humidity) of the buds.

Like other NTP methods, specific legislation for this decontamination process on foods does not currently exist to inform the regulations necessary for cannabis. Pampoukis et al. proposed a metadata schema to standardize NTP methods for decontamination to improve the use of data to inform future legal regulations (Pampoukis et al., 2025). All of the core elements within the schema, including the plasma source, medium, target, and diagnostics, will be important to consider when treating cannabis. Specifically, for PPA, gas properties, such as the gas flow rate, temperature, and humidity, are important to consider as a medium because they affect the plasma chemistry and the resulting active species (Pampoukis et al., 2025). Standardizing the parameters for understanding the data on NTP decontamination of food and cannabis will make it possible to create legislation that will eventually lead to the possibility of this process being used in a safe, effective, and commercially viable manner.

## Conclusion

Cannabis is increasingly being used for medicinal and recreational purposes; nevertheless, it is subject to contamination as a natural product during its growth and processing. Microbiological contamination, particularly by pathogenic fungi and bacteria, poses risks to both the plant, affecting the yield and quality of the produce, and to people who consume cannabis, which may carry harmful pathogens. Some of these pathogens, such as *Aspergillus*, have been found to cause severe illnesses in consumers. Therefore, there are regulations on cannabis, particularly in medicine, for acceptable microbiological load limits, measured in terms of TAMC and TYMC. These limits are achieved by growing cannabis under proper GMP standards and by postharvest sanitation methods in an attempt to reduce the pathogens present on cannabis.

Cannabis is a sensitive product that is not suitable for decontamination methods that significantly increase its temperature, which affects its content of cannabinoids and/or terpenes and thereby its pharmacological and sensory properties, as well as methods that use chemical disinfectants due to the risk of lingering chemicals within the complex flower structure. Popular sanitation methods that are currently used on cannabis include gamma irradiation and electron beam irradiation. While these processes are proven to be effective at reducing the microbiological load, they present significant regulatory, cost, and customer approval challenges to the widespread acceptance of these methods. This is primarily due to skepticism around irradiation due to perceived consequences about the safety and quality of irradiated products. Some gaseous sanitation agent methods, including ozone and vacuum-water vapor-vacuum, are beginning to be applied in the cannabis industry, which may reduce this concern of using irradiation.

New and innovative technologies such as non-thermal plasma are therefore increasingly in research focus, as they offer the potential to achieve the microbial reduction required to ensure consumer safety while avoiding high temperatures and chemical disinfectants. These non-thermal plasma techniques are emerging as potential ways to sanitize cannabis, with the prospect of improved industrial feasibility and customer acceptance. One NTP method currently under research utilizes reactive nitrogen species within plasma-processed air to remove microbial contaminants. PPA has undergone laboratory testing for use in food applications that can help inform how it can be used on cannabis. Ozone and PPA treatment systems are currently being tested for the reduction of microbiological contaminants on cannabis specifically. These two methods appear to be the most promising methods, both generated with NTP techniques, because they take advantage of reactive species in a gaseous state that can possibly diffuse into the complex flower structure, destroying hard-to-reach microbiological contaminants.

The main challenge in developing a new germ reduction process is to achieve a microbial reduction that is comparable to existing germ reduction processes. Moreover, it must be ensured that the quality of the cannabis flowers meets the criteria of the release specifications. Not only must quantitative parameters such as cannabinoid and terpene contents be preserved, but organoleptic properties such as overall appearance, color, and odor must also remain unchanged. Therefore, future research must place a stronger emphasis on comprehensive stability studies to investigate potential long-term effects of novel decontamination methods. These studies should include microbial load, active ingredient content, and especially organoleptic properties, as the latter are closely linked to customer approval. Color changes require particular attention, as prior studies on dry food matrices treated with plasma-processed air have reported browning or pigment degradation, potentially linked to the destruction of carotenoids or chlorophyll. Additionally, terpene degradation and general plant degradation processes should be systematically analyzed. Based on such findings, treatment parameters, especially for ozone and PPA, will likely need to be optimized to balance effective microbial reduction with preservation of product quality, as adverse quality effects in plasma-treated foods have been associated with the most aggressive antimicrobial conditions.

For ozone treatment in particular, it remains inconclusive whether microbial efficacy is consistently associated with adverse quality changes in medicinal plants. Consequently, future data generated under optimized treatment parameters are required to clarify whether ozone can achieve sufficient microbial reduction on cannabis without compromising quality or consumer acceptance. In addition, NTP systems that do not rely on consumable chemical agents, such as hydrogen peroxide, may be advantageous for both industry and consumers. Processes such as PPA, which are generated from compressed air and do not require costly chemicals or noble gases, may therefore offer improved commercial viability and customer acceptance.

As is common for novel technologies applied to new industrial contexts, regulatory guidelines and standards for NTP-based decontamination are currently lacking. Therefore, the establishment and adoption of standardized metadata schemas for NTP applications are critical. Given the heterogeneity of non-thermal plasma sources and application conditions, including plasma generation, treatment medium, target material, diagnostics, gas composition, flow rate, temperature, and humidity, standardized reporting is essential to improve data quality and comparability. Recent metadata schemas, represent an important first step and may be adapted for cannabis flower applications in the future, provided they are widely adopted by the research community.

While this review primarily focuses on bacterial and fungal contamination as the most immediate risks, cannabis plants are also affected by other biological contaminants, including viruses, viroids, oomycete., and parasitic nematodes. The efficacy of ozone and NTP-based methods against these contaminant groups on cannabis flowers remains largely unexplored and should be investigated to establish a comprehensive safety profile.

In summary, non-thermal plasma methods that are known to efficiently inactivate microorganisms in food products may be used for cannabis sanitation. Ozone and PPA, in particular, present interesting alternatives to the irradiation methods currently used on cannabis as potentially effective, non-thermal, and residue-free sanitation processes. Another advantage of ozone and PPA is the possibility to treat cannabis locally, instead of sending it to a centralized facility, which means that even smaller growers can decontaminate their product. Current research indicates that up-scaling these processes is also likely possible if larger batches need to be treated. Ultimately, aspects such as the intensity of decontamination, the type of microorganism, the conditions of the treatment environment, safety, and economic factors must be taken into account to identify the best application of non-thermal plasma techniques. As further research progresses, the development of standardized regulatory and legislative frameworks will be a key step toward final industrial implementation. The goal of introducing novel decontamination techniques is to guarantee that medicinal cannabis, as well as recreational cannabis, maintains high safety and quality needs under feasible and effective industrial decontamination processes.

## Data Availability

Not applicable, as it is a review article and no datasets were generated.

## Abbreviations

WHO: World Health Organization
THCA: Tetrahydrocannabinolic acid
THC: Tetrahydrocannabinol
GMP: Good Manufacturing Practice
GACP: Good Agricultural and Collection Practice
CBD: Cannabidiol
CBDA: Cannabidiolic acid
FDA: Food and Drug Administration
PGPB: Plant growth-promoting bacteria
TAMC: Total aerobic microbial count
TYMC: Total yeast and mold count
CFU: Colony forming units
GRAS: Generally recognized as safe
NTP: Nonthermal plasma
DBD: Dielectric barrier discharge
RNS: Reactive nitrogen species
PPA: Plasma processed air
